# Correlation of T Cell Subsets and Hypercholesterolemia of the Donor and Its Association with Acute Graft-versus-Host Disease

**Published:** 2019-10-01

**Authors:** MM Rivera-Franco, Eucario León-Rodríguez, Diana Gómez-Martín

**Affiliations:** 1 Stem Cell Transplantation Program, Department of Hematology and Oncology, Instituto Nacional de Ciencias Medicas y Nutricion Salvador Zubiran, Mexico City, Mexico; 2 Department of Immunology and Rheumatology, Instituto Nacional de Ciencias Médicas y Nutrición Salvador Zubirán, Mexico City, Mexico

**Keywords:** Tregs, Acute graft-versus-host disease, Hypercholesterolemia, Allogeneic hematopoietic stem cell transplantation

## Abstract

**Background: **Acute graft-versus-host disease (aGVHD) is an important cause of death following allogeneic hematopoietic stem cell transplantation (allo-HSCT). The association between cholesterol and aGVHD was previously described potentially, resulting from pro-inflammatory responses associated with hypercholesterolemia. The aim of this study was to correlate T cell subsets in donor bone marrow (BM) samples with their levels of cholesterol and associate these results with recipients who developed aGVHD and those who did not.

**Materials and Methods: **A prospective study was performed in 39 donor samples. T cell subsets were analyzed by flow cytometry.

**Results: **Eleven (28%) donors had hypercholesterolemia. Donor samples with hypercholesterolemia had less Tregs compared to donors with normal levels of cholesterol (22.69 (IQR=30.6) cells/µL vs 52.62 (IQR=44.68) cells/µL, p=0.04). Among all individuals in the cohort, aGVHD was observed in 21%: 36% from donors with hypercholesterolemia versus 14% from donors with normal levels of cholesterol.

**Conclusion: **As we described the association between hypercholesterolemia and diminished Tregs, our results might suggest that normalizing the levels of total cholesterol in the donor, prior performing allo-HSCT, might be an effective approach to diminish the risk of the receptor to develop aGVHD.

## Introduction

 Worldwide, the rate of allogeneic hematopoietic stem cell transplantation (allo-HSCT) is increasing^1^^,^^2^ despite its association with increased morbidity and non-relapse mortality (NRM). Among the complications, for instance, hypercholesterolemia has been reported to be a consequence of allo-HSCT^3^^-^^5^. Nonetheless, acute graft-versus-host disease (aGVHD) remains the second leading cause of death following this procedure ^6^. In this context, some authors have reported a decreased frequency of aGVHD using statins as a result of their anti-inflammatory effects^7^^-^^11^. However, the direct association between high cholesterol and GVHD was not described until last year when we reported that hypercholesterolemia in the recipients, donors, or both, prior allo-HSCT, was associated with increased frequency of this post-transplant complication^12^. This phenomenon could be potentially explained because hypercholesterolemia is associated with pro-inflammatory responses, thus potentially leading to increased aGVHD. Moreover, it is known that depletion of regulatory T cells (Tregs) is associated with worsening of GVHD as first described by Taylor et al.^13^, thus Tregs are essential for the induction of tolerance to allo-antigens, which has been widely demonstrated in murine models^14^ while preserving the beneficial graft-versus-leukemia effect^15^. Furthermore, Tregs have the immunosuppressive ability to regulate of immune responses^16^. Rezvani et al.^17^ found that increased frequencies of CD4^+^Foxp3^+^ Tregs in the peripheral blood of the donor negatively correlated with the incidence of GVHD in the graft recipient. Subsequent studies have confirmed this correlation in the recipients of HLA-identical sibling and unrelated donor stem cell grafts^18^^,^^19^, indicating that infused donor Tregs in graft contents appear to lessen the severity of GVHD. 

In order to further investigate a potential mechanism of the pro-inflammatory effects of hypercholesterolemia potentially causing aGVHD, the aim of this study was to correlate Tregs in donor bone marrow (BM) samples with their levels of cholesterol and associate these results with recipients who developed aGVHD and those who did not.

## MATERIALS AND METHODS

**Patients, donors, and data**

A prospective analysis was performed in 39 donor bone marrow (BM) samples collected from March 2002 to April 2017. Data (demographic and clinical characteristics) were obtained from a prospectively created institutional HSCT database or retrospectively from the institutional medical records. Exclusion criteria included incomplete laboratory information, cord blood transplants, engraftment failure or 30-day mortality. 

**Stem cells harvesting**

Twenty-three bone marrow samples collected from 2002 to 2014 were thawed and then (20 mL) immediately processed and analyzed. These samples were derived from the harvesting of HSCs collected by multiple aspirations of the iliac crests in an operating room under spinal anesthesia 2 hours prior performing the HSCT. G-BM was obtained by administering G-CSF (10μg/kg/day) to donors, 3 days prior the collection and the day of the collection (4 days in total). The volume of harvest was adapted to the recipients’ body weight (BW) (maximum 15 ml/kg donors’ BW). For PBSC, HSCs were collected by apheresis with the administration of G-CSF for 5 days. 

**Stem cell transplantation**

Most patients with hematological malignancies received reduced BUCY2 (busulfan 12 mg/kg, ORAL and cyclophosphamide 80mg/kg, IV)^16^ which is a reduced intensity conditioning regimen (RIC), the rest received other myeloablative regimens (MAC). Patients with aplastic anemia received antithymocyte globulin (ATG) and/or cyclophosphamide-based conditioning regimens. Methotrexate (MTX) and cyclosporine A (CSA) were given for GVHD prophylaxis. MTX was administered IV, 15mg/m^2^ day +3, and 10mg/m^2 ^during days +6 and +11. None of the patients received MTX on day +1^17^. CSA was administered IV, 1.5mg/kg/12 hours, during day -1 and adjusted according to serum levels (200-300 ng/μl) until 2005. Afterwards, CSA was administered orally (IV presentation was withdrawn from the market), 10mg/kg on day -1, and 5 mg/kg starting day 0 and adjusting according to therapeutic monitoring. CsA was maintained for 4 months post-transplant (8 months in aplastic anemia), and was subsequently reduced weekly (10%) until suspended unless development of GVHD. Antimicrobial prophylaxis and supportive therapy were given according to Institutional guidelines. Patients were discharged when engraftment occurred and in the absence of infections or complications, and follow-up was performed in the out-patient clinic.

**T Cells Subsets Isolation**

BM mononuclear cells (BMMCs) were obtained by density-gradient centrifugation (Lymphoprep, Oslo, Norway). BMMCs were stained with anti-CD4-FITC (BD Biosciences, San Jose, CA). Intracellular staining was also performed. After fixation and permeabilization (Cytofix/Cytoperm Kit, BD Biosciences, San Jose, CA), cells were stained with either anti-IL-17A-PE, anti-IFNγ-APC, anti-IL4 PECy7 or anti-FOXP3-PE (BD Pharmingen, San Jose, CA). Data were collected on a LSR Fortessa cytometer (BD Biosciences, San Jose, CA) and analyzed using FlowJo (version 10, Tree Star Inc., Ashland, OR). The absolute count of T cells was calculated accounting the percentages obtained by flow cytometry and both leukocyte and lymphocyte BM absolute counts. 

**Endpoints and definitions**

Total cholesterol in the recipient and the donor was documented approximately 1 month prior performing HSCT as part of the pre-transplant workup. High cholesterol was considered as total cholesterol ≥ 200mg/dL according to international guidelines^20^. NIH criteria were used to diagnose and evaluate severity of acute GVHD ^21^.

**Statistical analysis**

Continuous variables were described by the median and interquartile range using the frequency analysis. Categorical variables were described by frequencies and percentiles. Comparison between variables was made by means of Student’s t test or one-way ANOVA. Categorical variables were compared with the chi-square or Fisher’s exact test. SPSS v.21 (IBM, Chicago, IL) was used. Two-sided p-values of <0.05 were considered significant.

## Results

 Thirty-nine donor bone marrow samples were analyzed as described in Table 1. Of 39 donors, 11 (28%) had hypercholesterolemia and 28 (72%) had normal levels of cholesterol. 

Patients having donors with normal levels of cholesterol were younger than patients with whose donors presented hypercholesterolemia. All patients had a matched-related donor. The most common source was G-BM (n=28, 72%), followed by SS-BM (n=10, 26%). Gender disparity was observed in 44%. Acute leukemia was the most frequent underlying diseases (n=14, 36%), followed by aplastic anemia (n=9, 23%). Most patients received myeloablative or reduced intensity conditioning regimens (77%). Median CD34+ cells was similar between both groups. 

Among all cohorts, acute GVHD was observed in 8 recipients (21%): 4 recipients (36%) from the 11 donors with hypercholesterolemia versus 4 recipients (14%) from the group of donors with normal levels of cholesterol (n=28). Across all cohorts, indistinctively from the level of cholesterol, acute GVHD was characterized by a predominant pro-inflammatory profile with increased Th17 cells (1051.35 (IQR=79.76) cells/µL vs 530.94 (IQR=18.78) cells/µL, p=0.03), and although this increase was not statistically significant, a pattern of deficient suppressor response with lower Tregs (26.88 (IQR=143.94) cells/µL vs 47.21 (IQR=44.25) cells/µL, p=0.7) was observed.

**Figure 1:**
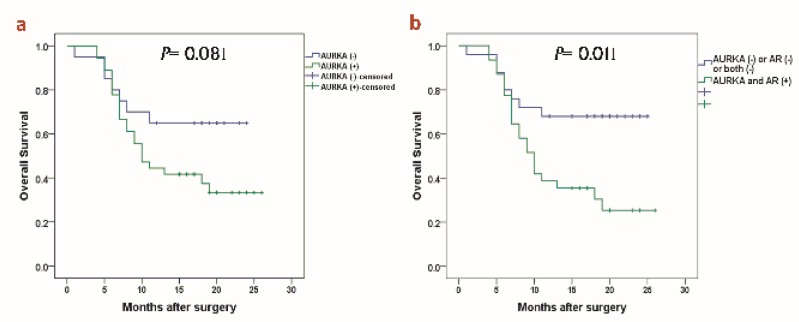
Tregs in donor bone marrow samples according to total cholesterol levels

As depicted in Table 2, donors with hypercholesterolemia presented higher levels of T cells subsets compared to donors with normal levels of cholesterol, showing statistically significant differences in Th1 and Tregs. In this context, donor samples with hypercholesterolemia showed a decreased suppressive phenotype characterized by diminished Tregs, compared to donors with normal levels of cholesterol (22.69 (IQR=30.6) cells/µLvs52.62 (IQR=44.68) cells/µL, p=0.04) (Figure 1).

**Table 1:** Patient demographic and clinical characteristics according to donor cholesterol level

** Cholesterol level**		
Characteristics	< 200mg/dL	≥ 200mg/dL
Number	28	11
Patient gender Male Female	15 (54) 13 (46)	7 (64) 4 (36)
Patients’ median age	23 (IQR=13)	43 (IQR=15)
Disease Acute leukemias Aplastic anemia Chronic myeloid leukemia Myelodysplastic syndrome Others	8 (29) 6 (21) 5 (18) 5 (18) 4 (14)	6 (55) 3 (27) 0 1 (9) 1 (9)
HLA status Matched related donor Matched unrelated donor	28 (100) 0	11 (100) 0
Gender disparity None Female receptor, male donor Male receptor, female donor	13 (46) 7 (25) 8 (29)	9 (82) 1 (9) 1 (9)
Conditioning regimen Myeloablative/RIC Non-myeloablative	22 (79) 6 (21)	8 (73) 3 (27)
Source G-BM SS-BM PBSC	23 (82) 5 (18) 0	5 (45.5) 5 (45.5) 1 (9)
Median CD34+ x (10^6^/kg)	1.89 (IQR=0.97)	1.87 (IQR=1.5)
GVHD Acute Grades I-II Grades III-IV	4 (14) 2 (4) 2 (4)	4 (36) 1 (9) 3 (27)

Abbreviations**: **G-BM: G-CSF-primed bone marrow; MDS: Myelodysplastic syndrome; PBSC: Peripheral blood stem cells;

RIC: Reduced intensity conditioning; SS-BM: Steady-state bone marrow

**Table 2:** T-cell profile according to cholesterol levels in donor BM samples

	**Cholesterol level**		
**T- cell profile (cells/µL)**	**< 200mg/dL**	**≥ 200mg/dL**	**p**
Th 1 Th 2 Th17 Tregs	655.24 (IQR=802.3) 54.04 (IQR=47.19) 14.53 (IQR=27.06) 52.62 (IQR=44.68)	368.74 (IQR=255.4) 23.07 (IQR=29.1) 11.30 (IQR=18.7) 22.69 (IQR=30.6)	0.04 0.18 0.18 0.04

## Discussion

 This is the first study associating T cells subsets with cholesterol levels in the context of allo-HSCT. Our results showed that bone marrow samples of donors with normal levels of cholesterol displayed a more suppressive phenotype with increased percentages of Th2 and Tregs, compared to donors with hypercholesterolemia. Acute GVHD was higher in patients whose donors had hypercholesterolemia and we demonstrated that BM samples of the latter showed a less favorable immunologic profile characterized by diminished Tregs. The proportions of Th1 and Th17 cells were similar despite cholesterol levels.

Tregs are critically involved in the maintenance of immune tolerance to self and in the control of immune and autoimmune responses^22^. GVHD is characterized by the development of autoimmune manifestations as a result of the loss of tolerance in this context, thus a deficiency in Tregs reconstitution plays a critical role in its pathophysiology. Overall, T cells are receiving growing interest as major regulators of certain metabolic diseases such atherosclerosis. Guasti et al.^23^ investigated if the percentage of CD4 + cell subsets was expressed differently between dyslipidemic patients and healthy controls and whether treatment with atorvastatin could be associated with cell changes in expression and functional response. The authors found that although at the beginning Tregs were similar between the two groups, these cells were significantly correlated with total cholesterol, showing a decrease in patients with dyslipidemia at the second follow-up visit^23^. Furthermore, it has been described that prolonged hypercholesterolemia impairs the function of Tregs, and cholesterol lowering therapies could induce dynamic and beneficial changes in T cell subsets^24^^.^

We acknowledge that the small sample size represents a limitation of this study and larger studies are required; however, our results represent an approach to further understand one of the potential mechanisms of hypercholesterolemia increasing aGVHD.

## CONCLUSION

 In conclusion, as aGVHD substantially contributes to the morbidity and mortality after allo**-**HSCT, more effective prevention strategies are needed, and after potentially explaining the association between total cholesterol levels, inflammation, and Tregs, our results might suggest that normalizing the levels of total cholesterol in the donor, prior to allo-HSCT, might be an effective approach to diminish the risk of the receptor to develop aGVHD, especially among Mexican or American populations where dyslipidemia represents a public health concern^25^^26^.

## References

[1] Jaimovich G, MartinezRolon J, Baldomero H (2017). Latin America: the next region for haematopoietic transplant progress. Bone Marrow Transplant.

[2] Niederwieser D, Baldomero H, Szer J (2016). Hematopoietic stem cell transplantation activity worldwide in 2012 and a SWOT analysis of the Worldwide Network for Blood and Marrow Transplantation Group including the global survey. Bone Marrow Transplant.

[3] Griffith ML, Savani BN, Boord JB (2010). Dyslipidemia after allogeneic hematopoietic stem cell transplantation: evaluation and management. Blood.

[4] Joukhadar R, Chiu K (2012). Severe hypercholesterolemia in patients with graft-vs-host disease affecting the liver after stem cell transplantation. Endocr Pract.

[5] Marini BL, Choi SW, Byersdorfer CA (2015). The Treatment of Dyslipidemia in Allogeneic Hematopoietic Stem Cell Transplant Patients. Biol Blood Marrow Transplant.

[6] Nassereddine S, Rafei H, Elbahesh E (2017). Acute Graft Versus Host Disease: A Comprehensive Review. Anticancer Res.

[7] Broady R, Levings MK (2008). Graft-versus-host disease: suppression by statins. Nat Med.

[8] Rotta M, Storer BE, Storb RF (2010). Donor statin treatment protects against severe acute graft-versus-host disease after related allogeneic hematopoietic cell transplantation. Blood.

[9] Rotta, Storer BE, Storb R (2010). Impact of recipient statin treatment on graft-versus-host disease after allogeneic hematopoietic cell transplantation. Biol Blood Marrow Transplant.

[10] Shimabukuro-Vornhagen A, Liebig T, Bergwelt-Baildon M (2008). Statins inhibit human APC function: implications for the treatment of GVHD. Blood.

[11] Zeiser R, Youssef S, Baker J (2007). Preemptive HMG-CoA reductase inhibition provides graft-versus-host disease protection by Th-2 polarization while sparing graft-versus-leukemia activity. Blood.

[12] Rivera-Franco MM, León-Rodríguez E, Lastra-German IK (2018). Association of recipient and donor hypercholesterolemia prior allogeneic stem cell transplantation and graft-versus-host disease. Leuk Res..

[13] Taylor PA, Noelle RJ, Blazar BR (2001). CD4 (+) CD25 (+) immune regulatory cells are required for induction of tolerance to alloantigen via costimulatory blockade. J Exp Med.

[14] Chen X, Vodanovic-Jankovic S, Johnson B (2007). Absence of regulatory T-cell control of TH1 and TH17 cells is responsible for the autoimmune-mediated pathology in chronic graft-versus-host disease. Blood.

[15] Edinger M, Hoffmann P, Ermann J (2003). CD4+CD25+ regulatory T cells preserve graft-versus-tumor activity while inhibiting graft-versus-host disease after bone marrow transplantation. Nat Med.

[16] Fontenot JD, Rasmussen JP, Williams LM (2005). Regulatory T cell lineage specification by the forkhead transcription factor foxp3. Immunity.

[17] Rezvani K, Mielke S, Ahmadzadeh M (2006). High donor FOXP3-positive regulatory T-cell (Treg) content is associated with a low risk of GVHD following HLA-matched allogeneic SCT. Blood.

[18] Wolf D, Wolf AM, Fong D (2007). Regulatory T-cells in the graft and the risk of acute graft-versus-host disease after allogeneic stem cell transplantation. Transplantation.

[19] Pabst C, Schirutschke H, Ehninger G (2007). The graft content of donor T cells expressing gamma delta TCR+ and CD4 + foxp3+ predicts the risk of acute graft versus host disease after transplantation of allogeneic peripheral blood stem cells from unrelated donors. Clin Cancer Res.

[20] Nayor M, Ramachandran S, Vasan RS (2016). Recent Update to the US Cholesterol Treatment Guidelines: A Comparison With International Guidelines. Circulation.

[21] Vigorito AC, Campregher PV, Storer BE (2009). Evaluation of NIH consensus criteria for classification of late acute and chronic GVHD. Blood..

[22] Galgani M, De Rosa V, La Cava A (2016). Role of Metabolism in the Immunobiology of Regulatory T Cells. J Immunol.

[23] Guasti L, Maresca AM, Schembri L (2016). Relationship between regulatory T cells subsets and lipid profile in dyslipidemic patients: a longitudinal study during atorvastatin treatment. BMC Cardiovasc Disord.

[24] Maganto-García E, Tarrio ML, Grabie N (2011). Dynamic changes in regulatory T cells are linked to levels of diet-induced hypercholesterolemia. Circulation.

[25] Aguilar-Salinas CA, Gómez-Pérez FJ, Rull J (2010). Prevalence of dyslipidemias in the Mexican National Health and Nutrition Survey 2006. Salud Publica Mex..

[26] Carroll MD, Fryar CD, Kit BK (2015). Total and high-density lipoprotein cholesterol in adults: United States,2011–2014. NCHS Data Brief.

